# Ultrafast laser filament-induced fluorescence for detecting uranium stress in *Chlamydomonas reinhardtii*

**DOI:** 10.1038/s41598-022-21404-z

**Published:** 2022-10-13

**Authors:** Lauren A. Finney, Patrick J. Skrodzki, Nicholas Peskosky, Milos Burger, John Nees, Karl Krushelnick, Igor Jovanovic

**Affiliations:** 1 Department of Nuclear Engineering and Radiological Sciences, University of Michigan, Ann Arbor, MI 48109 USA; 2 Gérard Mourou Center for Ultrafast Optical Science, University of Michigan, Ann Arbor, MI 48109 USA

**Keywords:** Light responses, Plant stress responses, Environmental sciences, Imaging and sensing, Optical spectroscopy

## Abstract

Plants and other photosynthetic organisms have been suggested as potential pervasive biosensors for nuclear nonproliferation monitoring. We demonstrate that ultrafast laser filament-induced fluorescence of chlorophyll in the green alga *Chlamydomonas reinhardtii* is a promising method for remote, in-field detection of stress from exposure to nuclear materials. This method holds an advantage over broad-area surveillance, such as solar-induced fluorescence monitoring, when targeting excitation of a specific plant would improve the detectability, for example when local biota density is low. After exposing *C. reinhardtii* to uranium, we find that the concentration of chlorophyll a, chlorophyll fluorescence lifetime, and carotenoid content increase. The increased fluorescence lifetime signifies a decrease in non-photochemical quenching. The simultaneous increase in carotenoid content implies oxidative stress, further confirmed by the production of radical oxygen species evidence in the steady-state absorption spectrum. This is potentially a unique signature of uranium, as previous work finds that heavy metal stress generally increases non-photochemical quenching. We identify the temporal profile of the chlorophyll fluorescence to be a distinguishing feature between uranium-exposed and unexposed algae. Discrimination of uranium-exposed samples is possible at a distance of $\sim$35 m with a single laser shot and a modest collection system, as determined through a combination of experiment and simulation of distance-scaled uncertainty in discriminating the temporal profiles. Illustrating the potential for remote detection, detection over 125 m would require 100 laser shots, commensurate with the detection time on the order of 1 s.

## Introduction

The response of plants to stress and toxicity has been the subject of numerous studies. Plant health monitoring is relevant to many applications including agriculture^1^, pollution monitoring^2^, biosensor development^3–5^, biofuels^6^, and remediation efforts for nuclear accidents^7,8^. The majority of prior studies focused on the effect of natural stresses such as drought, or the exposure to heavy metals associated with industrial pollution, such as lead, cadmium, and arsenic^9–12^. There has been recent interest in using biota as biosensors for uranium (U) contamination in the environment. In addition to area monitoring in the case of accidental nuclear releases, it has also been suggested that such biosensors may enable large-area surveys for detecting nuclear proliferation activities. Future progress in this field critically depends on the understanding of the specificity of plant’s response to U contamination and the optimal method to detect this response, especially over long distances^13–18^. One highly relevant proliferation scenario that includes a pathway for U contamination is clandestine enrichment of U^19^. For example, the centrifuge process employs UF$_6$ gas to alter the isotopic composition of naturally occurring U (consisting predominantly of $^{238}$U and $^{235}$U) to greater concentrations of $^{235}$U. Small quantities of UF$_6$ gas can escape from these facilities and interact with water in the atmosphere to form UO$_2$F$_2$, which can deposit in surrounding soils and water systems and be absorbed by or adsorbed onto plants.

Prior studies have explored the form of U that is most bioavailable across several species, such as algae^20^, *Arabidopsis thaliana*^14,21^, *Brassica juncea*^22^, and many others. In all cases it has been found that U in the form of the uranyl ion (UO$_2^{2+}$) is the most mobile in soil and roots and is the dominant form at slightly acidic pH’s, around 4.5–6.5. It has also been found that this ion chelates with other essential nutrients commonly found in plant soil, such as phosphates and carbonates^13,16^. In general, it has been found that U is toxic to plants, evidenced by the formation of radical oxygen species (ROS) such as H$_2$O$_2$, and that the proteins glutathione and ascorbate play an essential role in mitigating the effects of ROS damage to photo-sites within the photosynthetic chain^17,23^. The efficacy with which various algae species can remove U from water systems was investigated^20^, along with the effect of U exposure on the photosynthetic efficiency and function of algae^24–26^. It has been reported that microalgae can be highly resistant and adaptable to harsh growth conditions, such as extreme pollution from uranium mining^24^. While the uptake of and plant response to U has been investigated, signatures of U exposure that could be used for remote and rapid in-field detection have not been explored.

Two optical properties of plants have been of primary interest: (1) broadband reflectance^27^, which provides information on the water content, biomass, and pigment concentrations, and (2) fluorescence of chlorophyll a (Chl a), which can provide information on photosynthetic activity^28,29^. The three main methods that have been used to interrogate those properties are pulse-amplitude modulated (PAM) fluorometry, hyperspectral imaging through reflectance spectroscopy, and remote solar-induced fluorescence (SIF). Only the latter two methods are suitable for remote detection. The main drawback of these techniques is that solar radiation contributes significantly to the background, and weak signal is observed in areas of low plant density, particularly in the case of SIF.

A promising method for remote optical sensing is based on ultrashort-pulse laser filamentation^30^. Filamentation occurs as a result of the Kerr effect, where an intense laser pulse induces a change in the refractive index of the propagation medium that is nonlinearly dependent on the laser intensity. The change in refractive index results in beam self-focusing and, eventually, plasma formation in a confined columnar structure known as the filament. Self-focusing exhibits a threshold known as the critical power, $P_{cr} =C \lambda ^2/(8 \pi n_2 n_0)$^31^, where $\lambda$ is the laser wavelength, $n_2$ is the nonlinear refractive index, $n_0$ is the linear refractive index of the medium, and *C* is a constant dependent on the beam profile ($C=3.77$ for a Gaussian). Filamentation in air is an area of ongoing research for remote sensing through techniques such as laser-induced fluorescence^32^, filament-induced breakdown spectroscopy^33–36^, and LIDAR^37,38^. Laser-based excitation of chlorophyll in plants has been explored with nanosecond lasers, but femtosecond filament excitation of biota has yet to be reported. A special benefit when using ultrashort pulses is the ability to resolve rapid molecular dynamics, such as nonphotochemical quenching (NPQ) that occurs on the $\lesssim$100-ps scale and in-vivo Chl fluorescence (ChlF), which has a lifetime on the order of 1 ns. Here we demonstrate the first use of ultrafast laser filaments to excite and detect ChlF in the green alga *Chlamydomonas reinhardtii*. We also explore the changes in the ChlF parameters in healthy algae and U-stressed algae. We discuss the relationship of these changes to the pigment concentrations measured through the steady-state absorption spectra. We further discuss the influence of pigments on the ChlF parameters observable via filament-induced fluorescence (F-IF). These methods offer the prospect for adaptation into practical in-field plant stress and health monitoring applications.

## Results

### Filament excitation and fluorescence analysis

**Figure 1:**
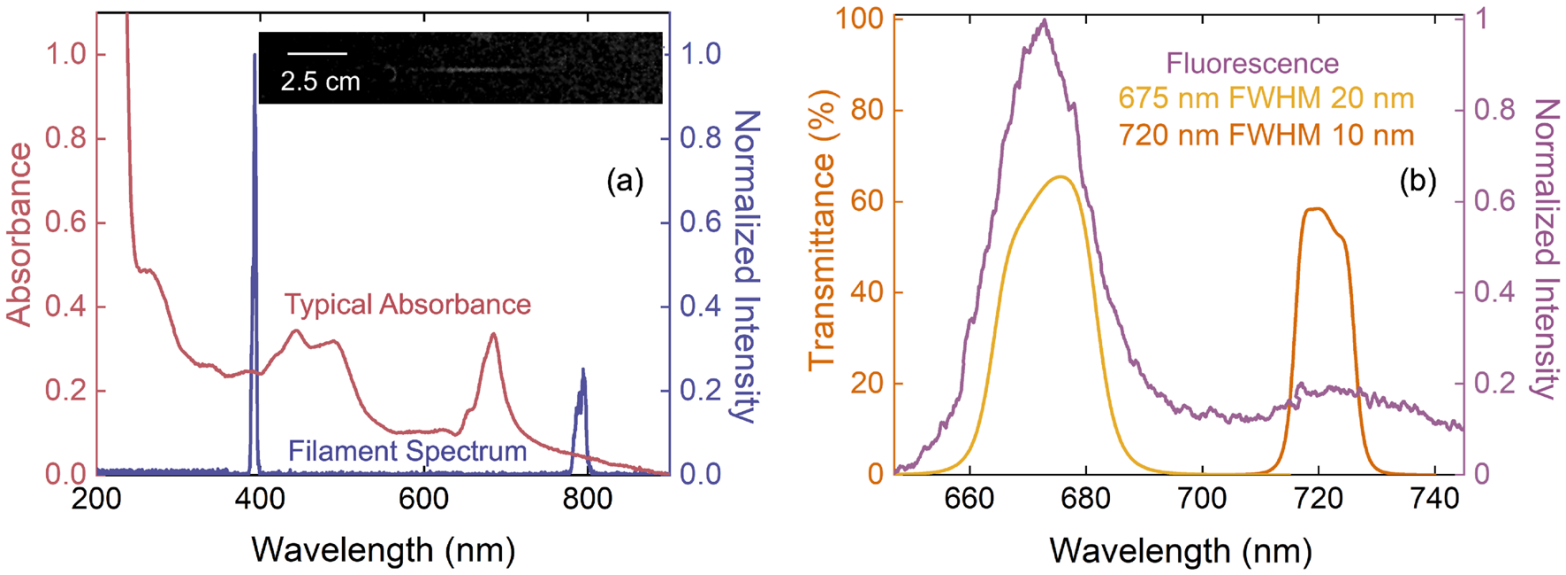
(**a**) Typical absorbance spectrum of *C. reinhardtii* and the filament excitation spectrum overlaid. The inset shows an image of the filament used for this experiment. (**b**) Example ChlF spectrum and the transmission spectra of the two spectral filters used for imaging.

**Figure 2:**
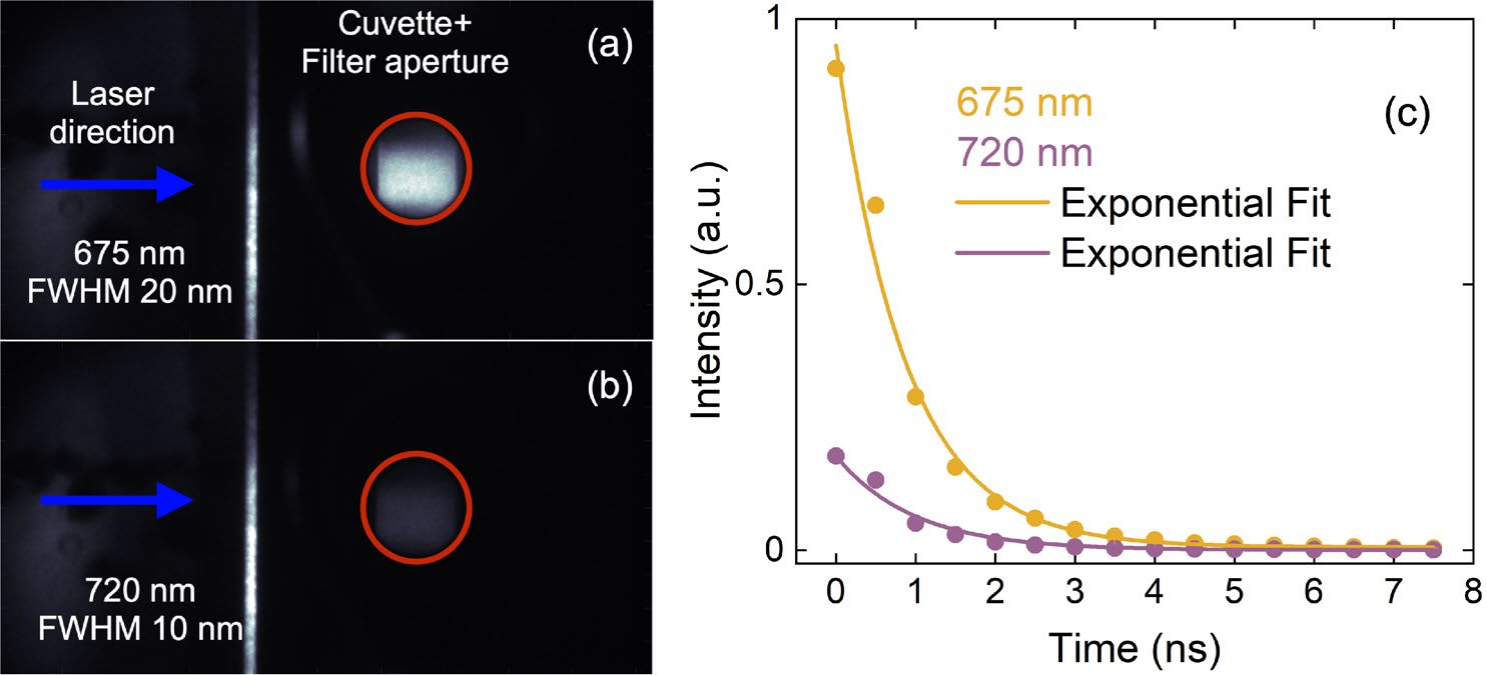
Fluorescence images recorded with the (**a**) 675-nm BPF and (**b**) 720-nm BPF. The red outlines define the region over which the total fluorescence intensity is integrated. Temporal profile of the two peaks (**c**), where the initial delay is 0 ns, the time step is 0.5 ns, and the gate width is 4 ns.

Figure 1a shows the filament excitation spectrum and a typical absorbance spectrum of the *C. reinhardtii* samples. The filament spectrum is measured with an integrating sphere and a fiber-coupled spectrometer (CCS200, Thorlabs) at the position where the samples are placed. The 395-nm peak from second-harmonic generation (SHG) of the fundamental laser wavelength can be seen. In Fig. 1b we show an example ChlF spectrum generated at room temperature and the transmission spectrum for each of the two bandpass filters (BPFs); each filter selects the emission from a single peak. When measured in-vivo, the two peaks are generated from different photosystems (PS) in the photosynthetic chain^39^. Photosystem II (PSII) primarily fluoresces at 675 nm, while PSI fluoresces at 720 nm. Fluorescence from each of the two peaks is measured separately to examine their relative response to U exposure. Example fluorescence images recorded with an EM-ICCD detector are shown in Fig. 2a,b for the 675 nm BPF and 720 nm BPF, respectively. To determine the total intensity, the images are cropped to contain only the aperture of the BPF, as depicted by the red outlines in Fig. 2a,b, and then integrated. A cuvette containing water is used as a background image, which is subtracted. We also recorded images at various delays with respect to the arrival of filament pulse to measure the ChlF lifetime, which is typically $\sim$700 ps–1 ns upon fs-laser excitation^40–43^. Here, we define the fluorescence lifetime as the constant of the exponential fit, *i.e.*, the time it takes the fluorescence intensity to drop to 1/e $\times$ its initial value. Figure 2c shows example time dependence of the fluorescence intensity in each peak; we use exponential fits to extract the lifetimes. These fluorescence lifetimes provide information on NPQ of ChlF by carotenoids (Car). In combination with the measurement of pigment concentration, it may be able to provide insight into the role of multi-functional pigments such as Car.

**Figure 3:**
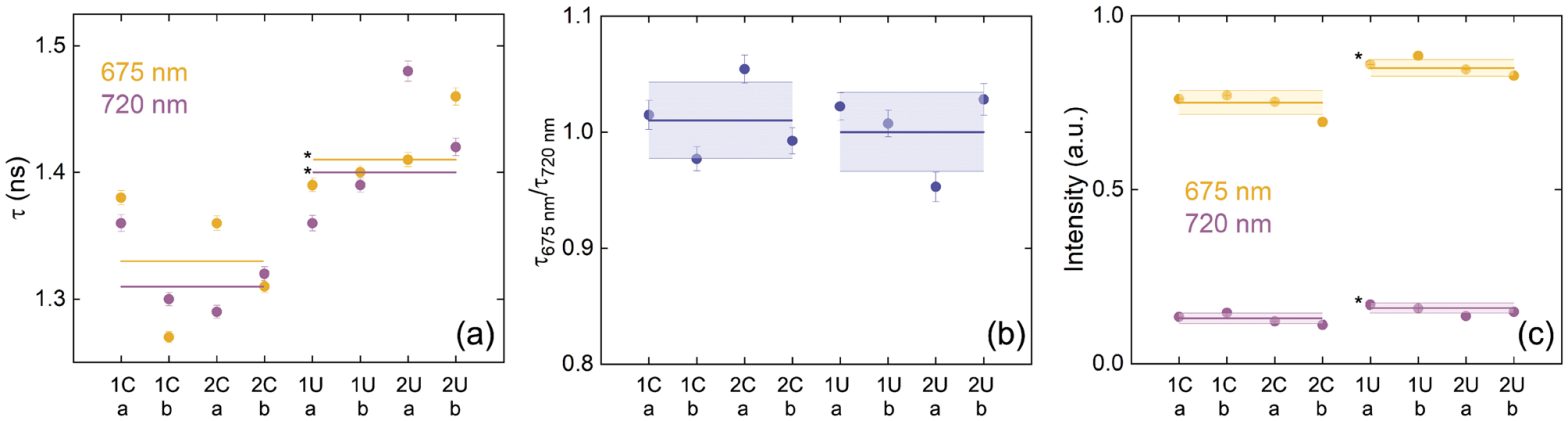
Lifetimes extracted for the (**a**) 675-nm peak and 720-nm peak; (**b**) the ratio of the lifetimes used to identify preferential disruption to PSII or PSI during stress; (**c**) time-integrated intensity for both peaks and all exposure conditions. The individual error bars correspond to the uncertainty in the exponential fit for (**a**), error propagated for the ratio of the lifetimes for (**b**), and error associated with the signal intensity assuming Poisson statistics for (**c**). The lines across the four data points represent the mean, while the shaded areas represent twice the standard error for four data points per condition. The control and U-exposed samples have the following means and twice the standard error, respectively: (**a**) at 675 nm – 1.33±0.04 and 1.41±0.04; at 720 nm – 1.31±0.04 and 1.40±0.06; (**b**) 1.01±0.04 and 1.00±0.04; (**c**) 0.75±0.04 at 675 nm, 0.13±0.02 at 720 nm and 0.85±0.02 at 675 nm, 0.16±0.02 at 720 nm. The asterisks (*) next to the mean uranium data denote that that t-test determined that they are statistically different from the mean of the control data.

Figure 3 presents the fluorescence lifetime, lifetime ratios, and overall intensities for each sample. The stock solutions are labeled by the number in front of a letter, where “C” stands for control and “U” stands for uranium. The labels “a” and “b” underneath denote each of the two samples per stock solution per condition. The thick lines on top of the four bars represents the mean value for four samples. In Fig. 3a,b, the mean fluorescence lifetime for both the 675-nm and 720-nm peaks are longer for the U exposed algae; however, in Fig. 3c the ratio of the lifetimes is approximately the same for the U exposed samples as for the control case. This implies that both photosystems are likely affected simultaneously by U stress. Lastly, the time-integrated signal intensity is greater for the U-exposed algae in both peaks as shown in Fig. 3d. An increase in total ChlF intensity may be due to increased Chl content. To confirm this observation, we next evaluate the pigment concentration for all samples using the UV-VIS-NIR absorption spectrum. We perform a t-test on the fluorescence data comparing the mean of the control samples to the uranium-exposed samples. Those found statistically different from the control mean at a 5% significance interval are labeled with an asterisk (*) in Fig. 3. The *p*-values for the 675 nm and 720 nm time-integrated intensities are 0.003 and 0.042, respectively. The *p*-values for the 675 nm and 720 nm lifetimes are 0.012 and 0.028, respectively. The *p*-value for $\tau _{\text {675~nm}}$/$\tau _{\text {720~nm}}$ is 0.693.

### Relationship between fluorescence and pigment content

**Figure 4:**
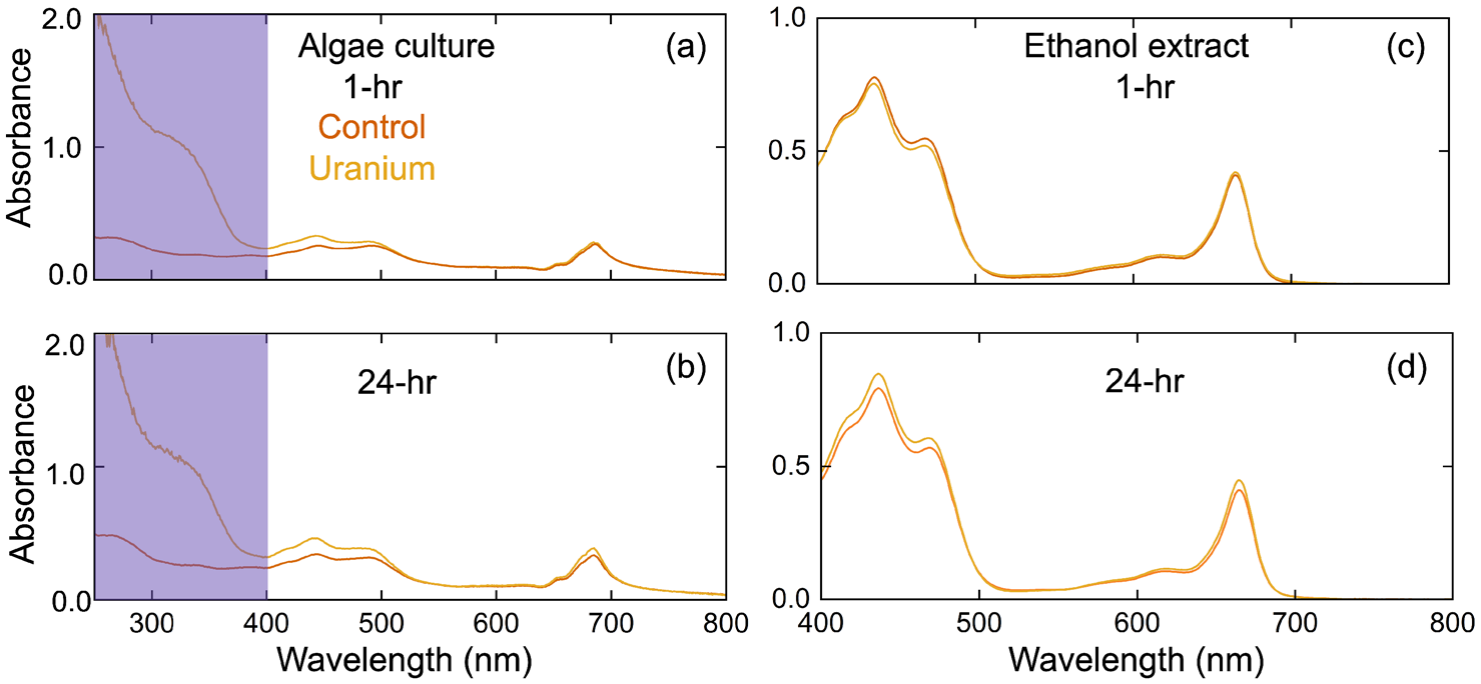
Representative absorbance spectra for the control and U-stressed algae at (**a**) 1-h and (**b**) 24-h exposure in growth media and the pigment extract in 95% ethanol for (**c**) 1-h and (**d**) 24-h. The spectrometer is zeroed to a cuvette containing water for (**a**,**b**), and to a cuvette containing 95% ethanol for (**c**,**d**).

The time-integrated fluorescence intensity at both 675 nm and 720 nm increases for the U-exposed algae, as shown in Fig. 3d. A potential reason for the increase in fluorescence emission is an increase in Chl content. Figure 4 presents representative absorbance spectra for the control and U-exposed algae after 1-h and 24-h exposures. In Fig. 4a, the algae suspended in growth media show that there is already a significant increase in absorption in the UV-region (250–400 nm) after 1 h for the U-exposed samples. Furthermore, the increased absorption persists through 24 h of exposure as seen in Fig. 4b. It is important to note that the absorbance for the control experimental media and U-containing media were measured and showed negligible absorbance to that presented by the algae suspended in culture in Fig. 4a,b.

**Figure 5:**
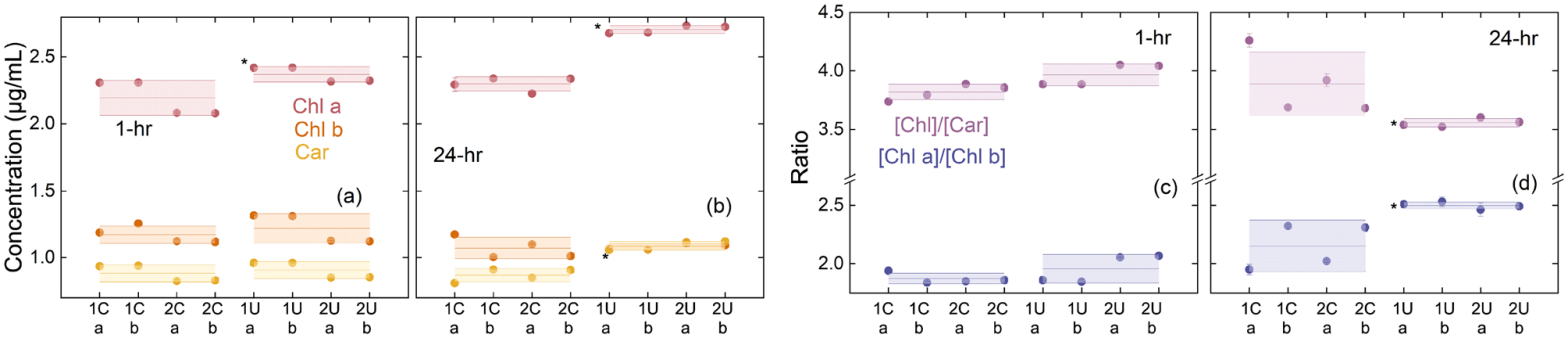
Pigment concentrations for each of the samples at (**a**) 1 h and (**b**) 24 h for the control and after exposure to U. The lines across each set of four data points are the mean for each exposure condition, and the shaded regions represent twice the standard error. The mean Chl a, Chl b, and Car concentrations ($\upmu$ g/mL), respectively, of the control samples for 1 h: 2.2, 1.2, and 0.9 & 24 h: 2.3, 1.1, 0.9. The mean Chl a, Chl b, and Car concentrations, respectively, of the U-exposed samples for 1 h: 2.4, 1.2, and 0.9 & 24 h: 2.7, 1.1, and 1.1. Relevant pigment ratios for each of the samples at (**c**) 1 h and (**d**) 24 h for the control and after exposure to U. The mean [Chl a]/[Chl b] and [Chl]/[Car] ratios of the control samples for 1 h: 1.8 and 3.7 & 24 h: 2.0 and 3.9. The mean [Chl a]/[Chl b] and [Chl]/[Car] ratios of the U-exposed samples for 1 h: 2.2 and 3.9 & 24 h: 2.5 and 3.6. In each, the mean and standard error are plotted over the 4 samples per exposure condition.The asterisks (*) next to the mean uranium data denote that the t-test found that they are statistically different from the mean of the control data.

To quantify the changes in the absorption spectra, we extract the pigments from the corresponding algae samples in 95% ethanol, and use the relations introduced by Lichtenthaler^44^ to determine the concentration of Chl a, Chl b, and total Car. Representative spectra for the U-exposed and unexposed algae pigment extracts at 1-h and 24-h exposures are shown in Fig. 4c,d. The results are shown in Fig. 5a,c for the 1-h exposure and in Fig. 5b,d for the 24-h exposure. We note that there is negligible change in the cell density within the duration of this experiment. The pigments are extracted 2:1 v/v for algae in media to 95% ethanol. The calculated concentrations are corrected for this difference in volume, and are reported in Fig. 5a,b as per mL of media or per 300,000 cells. The [Chl] notation represents the sum of the concentration of Chl a and Chl b. After 24 h of exposure, we see an increase in Chl a and Car concentration for the U exposed algae; furthermore, the [Chl]:[Car] ratio is lower for U-exposed samples than unexposed samples, having decreased from $\sim$3.9 to 3.6. This trend suggests that the change in concentration of Car increases more than the total change in Chl concentration when compared to the control case after 24-h of exposure. It is important to note that any changes in the photosynthetic apparatus, such as pigment and protein composition or pigment degradation under stress, can influence the accuracy of pigment quantification^45^. We perform a t-test on the pigment concentrations by comparing the mean of the control samples to the uranium-exposed samples. Those found statistically different from the control mean at a 5% significance are labeled with an asterisk (*) in Fig. 5. The *p*-values for the Chl a data at 1 h and 24 h are 0.009 and 9.4$\times$10$^{-5}$, respectively. The *p*-values for the Chl b data at 1 h and 24 h are 0.45 and 0.43, respectively. The *p*-values for the Car data at 1 h and 24 h are 0.50 and 9.8$\times$10$^{-4}$, respectively. The *p*-values for the [Chl]/[Car] data at 1 h and 24 h are 0.051 and 1.2$\times$10$^{-4}$, respectively. The *p*-values for the [Chl a]/[Chl b] data at 1 h and 24 h are 0.25 and 3.3$\times$10$^{-4}$, respectively.

**Figure 6:**
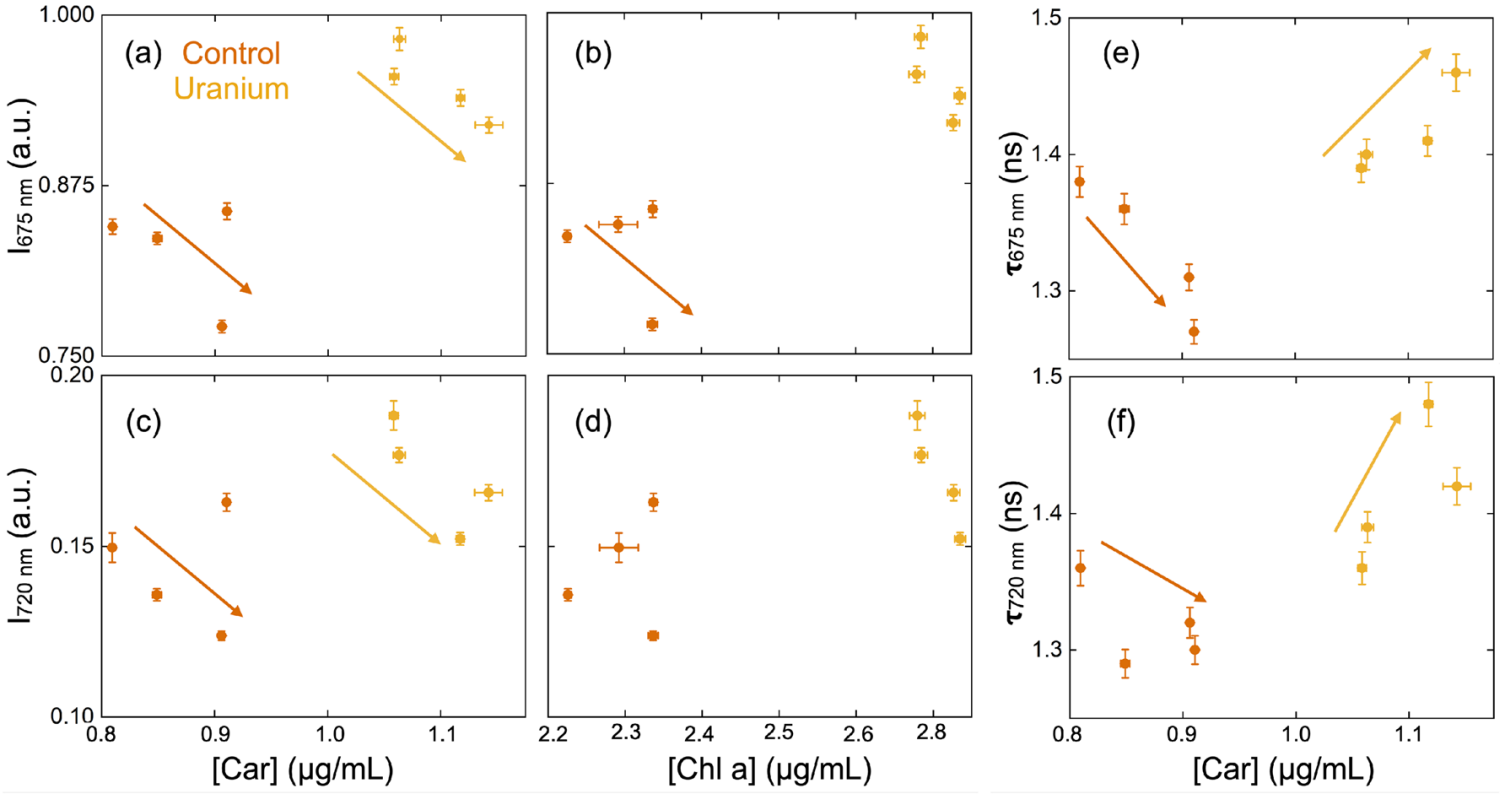
Time-integrated fluorescence intensity for the 675-nm peak as a function of (**a**) [Car] and (**b**) [Chl a]; similarly for the 720-nm peak in (**c**,**d**), respectively. The fluorescence lifetime as a function of [Car] for the (**e**) 675-nm peak and (**f**) 720-nm peak.

We next examine the dependence of the fluorescence lifetime and intensity on the pigment concentrations. In Fig. 6a,b, the 675 nm fluorescence intensity (I$_{675\text { nm}}$) is compared to the [Car] and [Chl a], respectively. First, for both the control and the U case there is a decrease in I$_{675\text { nm}}$ with increasing [Car]. Similarly, this trend is observed for the 720-nm peak in Fig. 6c,d. It is understood that Car assist in NPQ of ChlF to alleviate overly excited Chl from causing photosystem damage, which could explain the decrease in fluorescence intensity with increasing Car content. There is also a decrease in I$_{675\text { nm}}$ with increasing [Chl a] due to reabsorption of the fluorescence emitted by Chl a, which is less apparent for the 720-nm peak due to the absence of absorption by Chl a at this wavelength. There may be absorption of fluorescence by the cell wall; the turbidity level is related to the cell density, and therefore may also be related to the decrease in fluorescence measured.

One way to assess the level of NPQ is to observe changes in the fluorescence lifetime and the relationship to [Car]. When comparing $\tau _{675\text { nm}}$ to the [Car], the control samples follow the expected trend of decreasing lifetime, as seen in Fig. 6e. This is representative of increased NPQ, in which Chl a can dissipate of excess energy by transfer to Car. The other pathway of fluorescence quenching is through photosynthesis, or photochemical quenching (PQ). On the other hand, with the U-exposed samples, the increase in [Car] generally results in a longer $\tau _{675\text { nm}}$. This implies decreased NPQ; a similar trend is observed for the $\tau _{720\text { nm}}$ peak as shown in Fig. 6f. The fact that this correlation between [Car] and $\tau$ is apparent for both wavelengths suggests that neither PSII or PSI are predominantly attacked upon U exposure.

### Remote sensing of U-exposed algae

**Figure 7:**
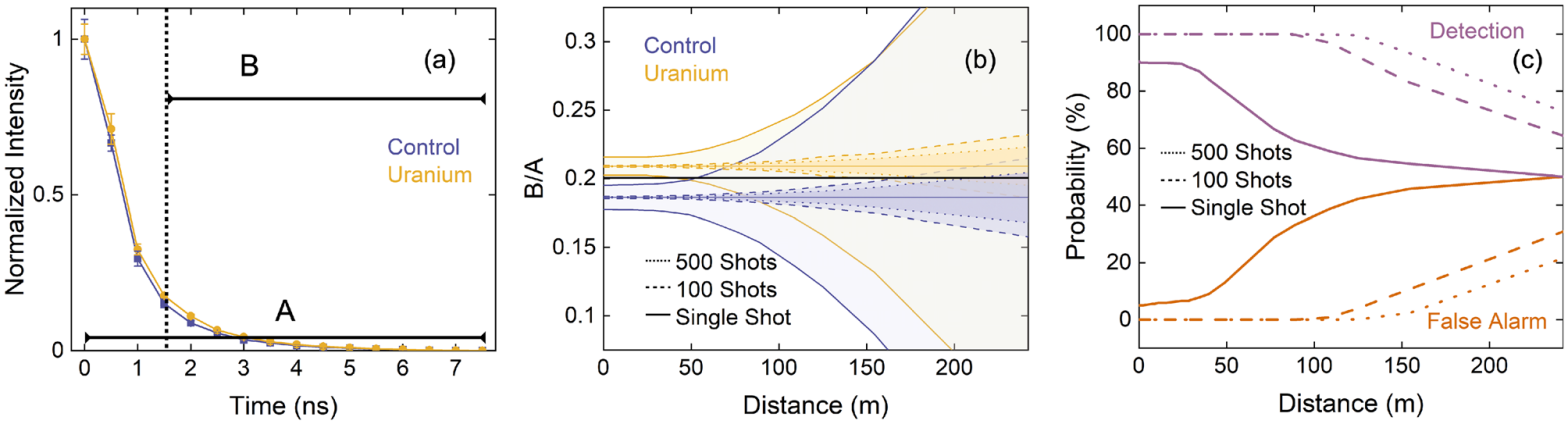
(**a**) Mean temporal profiles for the four control and four U-exposed algae samples, where the error bars represent 1-$\sigma$ standard deviation in the intensity at that time step. Segment “*B*” begins at 1.5 ns and “*A*” is the integral of the entire time profile. (**b**) *B*/*A* calculated from the measured temporal profiles at 0.15 m, and the distance-dependent uncertainties for measurements made with a single shot, 100 shots, and 500 shots. The black horizontal line denotes the critical limit set for determining the detection and false alarm probabilities. (**c**) Detection and false alarm probabilities as a function of distance determined from integrating the Gaussian distributions formed from the *B*/*A* and 1-$\sigma$ uncertainty calculated in (**b**).

Femtosecond laser filamentation enables remote excitation of optical signatures. In order to assess the potential for remote sensing using this approach, it is important to determine the distance at which these optical signatures can be measured. The ChlF lifetimes of the U-exposed algae samples are distinct from the controls; therefore, the shape of the temporal profile is promising for identification of the stress. We assess the performance of this discrimination method by comparing the temporal profiles recorded with the 675-nm BPF of the four control and U-exposed samples, as shown in Fig. 7a. The profiles are normalized so that the shape can be compared. In order to quantify this difference, we use the ratio of two segments of the profile, labelled “*A*” and “*B*” in Fig. 7a. We vary the start of segment “*B*” to maximize the distinction between the control and U-exposed profiles using a figure of merit (FOM) defined as follows: FOM$= (\mu _U - \mu _C)/(\sigma _C + \sigma _U)$, where $\mu _U$ and $\mu _C$ are the mean *B*/*A* integral ratio and $\sigma _U$ and $\sigma _C$ are the standard deviations of this ratio for the U-exposed and control algae, respectively. We find the optimal start time for the segment “*B*” is 1.5 ns. The detection probability is assessed by generating a normalized Gaussian distribution based on the mean *B*/*A* ratio and standard deviation for both the control and the U-exposed algae samples separately, setting a threshold of $B/A=0.206$ above which positive detection is assumed. At the distance of 0.15 m where the temporal profiles were recorded, this results in a single-shot detection probability of 90% and a false alarm probability of 5%.

To evaluate the potential to discriminate U-exposed algae from the control at greater distances, we assume an isotropic source and scale its intensity with distance. The *B*/*A* ratio has an experimentally determined uncertainty as the standard error of the measurements performed at 0.15 m for the four controls and four U-exposed samples. The absolute magnitude of this uncertainty scales with distance as it is dependent on the signal intensity. We also take into account the standard error in the ambient background that is subtracted from the signal, and which is measured in the laboratory environment with identical detector settings and is assumed to be independent of distance. Both of these uncertainties also scale with the number of averaged laser shots. We calculate the distance-dependent uncertainty in *B*/*A* for both the control and U-exposed algae samples for a single-shot measurement, 100 shots averaged, and 500 shots averaged, as shown in Fig. 7b. Above $\sim$35 m, we begin to see an increase in the standard error for the single-shot measurement. The impact of this change is also observed in Fig. 7c, where the detection probability remains constant below $\sim$35 m, and at greater distances the differences between the two profiles cannot be be discerned. The detection probability decreases with distance until it equals the false alarm rate. The false alarm and detection probabilities are calculated using the same critical limit set by the close-up measurement, which is denoted by the horizontal black line in Fig. 7b. The detection distance can be increased by accumulating the signal from multiple laser shots. We use the Currie criterion for minimum level of detection, where the false alarm rate is $\le$5% and the detection probability is $\ge$95%^46^. The criterion is met at 10 m for 2 shots, at 125 m with 100 shots, and at 150 m for 500 shots. With modern ultrafast laser systems having the ability to operate at $\sim$1-kHz repetition rate and assuming a comparable data acquisition system, the total measurement time of 1.6 s is required for 16 sample points across the time profile with accumulations of 100 shots. This analysis is based on the collection system used in the present experiment and the 500 $\upmu$ M U concentration and 24-h exposure period. Larger collection optics could improve the collection efficiency, and therefore the distance at which the signatures can be measured. Exposure that extends beyond the 24-h time-frame studied here could influence the magnitude of the algae stress response, resulting in a greater discrimination capability between the U-exposed and control algae.

Another important aspect of remote detection is the delivery of excitation energy. We found an input energy of 80 $\upmu$ J to be sufficient to generate a filament in the air over a distance of 125 m and excite ChlF in *C. reinhardtii* without causing noticeable damage to the sample. However, to generate a filament that will allow for confinement of the laser energy over much greater distances requires higher peak laser power. The formation of filaments on the order of kilometers away from the laser source has been demonstrated^47^, and filament ablation has been performed at a distance of 90 m using 800-nm driving wavelength and 250-mJ input energy^34^. Multiphoton ionization at 395 nm occurs at a significantly higher rate than at 800 nm, which results in rapid loss of energy with extended propagation through the filament plasma. To avoid this, one can use a telescopic focusing system, dispersion control, or a combination of both methods such that the filament formation distance can be controlled and formed much closer to the sample location^48^. Previous work exploring filamentation at various driving laser wavelengths and energies found that the energy loss after filamentation is about 25% at 0.5 mJ and 50% at an input energy for 4 mJ for filaments formed with *f*/40 focusing conditions^49^. Similar focusing conditions are used here for filament formation, but due to the low initial energy we can extrapolate the results from Ref.^49^ to find approximately 10% energy loss at 80 $\upmu$ J. Therefore $\sim$70 $\upmu$ J of energy reaches the algae samples in this experiment. Linear absorption through the atmosphere also needs to be accounted for in the extended delivery of laser energy. The predominant absorbing species in atmosphere for 395 nm light is NO$_2$, with a cross-section of 5$\times$10$^{-19}$ cm$^2$molecule$^{-1}$^50^. With a propagation distance of $\sim$100 m for detection with 100 laser shots and an estimated concentration of 6.5$\times$10$^{14}$molecules cm$^{-3}$^51^, the absorbance is 3.25, resulting in $\sim$0.06% of the light transmitted over 100 m through the atmosphere. To provide $\sim$80 $\upmu$ J for sample excitation, taking into account both the linear absorption and the filament energy loss of $\sim$50%, an initial energy of $\sim$260 mJ is required. Air turbulence and scattering can influence the initial energy needed to have sufficient energy at the sample position.

## Discussion

It is important to understand how U exposure affects fluorescence lifetime, which was determined to be a useful remotely discriminating signature, by means of NPQ and other physiological changes. Within the 24-h exposure, there does not seem to be a decrease in photosynthetic efficiency, as signified by the increase in Chl a content. A potential reason for increased Chl a content, despite being exposed to a toxic heavy metal like uranium, is the addition of nitrate to the growth media. Nitrate is a known nutrient to plants that promotes photosynthetic activity, and may simultaneously have an influence with uranium uptake. Further work can be done to investigate the influence of extra nitrate in the uranium-exposed samples, either through addition of the same quantity of a nitrate compound to the control or the use of an alternative uranium compound, such as uranyl chloride. However, the increase in [Car] is generally a sign of stress, in particular when the increase in [Car] is large such that [Chl]:[Car] is lower than that observed for the control case. A decrease in [Chl]:[Car] ratio is often observed in plant stress, especially associated with the production of ROS during drought stress or heavy-metal exposure^11^. In the case of drought, it has been observed that there is a substantial increase in NPQ due to the plants’ need to dissipate excess energy held by Chl a when it has minimal access to water to process, primarily at PSII. Previous work by Shibata et al.^42^ and Yamakawa et al.^40,41^ found that the time-integrated intensity and lifetime of the 675-nm peak was significantly decreased upon in-vivo fs-LIF of drought-tolerant moss as a result of increase NPQ. PSII is the main site involved in processing water in the photosynthetic chain^39^, and this is the hypothesized reason for the observed decrease in 675-nm peak fluorescence intensity and lifetime, while the 720-nm peak did not see as notable of a change.

Other than by disruption of the electron transport chain, there are several proposed mechanisms for U toxicity to plants, such as the substitution of Ca$^{2+}$ and Mn$^{2+}$ sites^52^. These chemical changes can influence the function of a photosystem. Since fs-LIF was able to identify a change in the fluorescence related to a specific photosystem under drought stress in previous experiments^40–42^, we compare the ratio of the lifetime of each peak in order to identify if one photosystem is undergoing a greater amount of quenching than the other for U exposure, as shown in Fig. 3c. We observe, however, that despite having overall increased fluorescence lifetimes upon U exposure, the $\tau _{\text {675~nm}}$/$\tau _{\text {720~nm}}$ ratio is consistent with the control case. This may mean that one photosystem is not being affected more than another within the timeframe of this study. This does not necessarily indicate that U is not detrimentally influencing the electron transport chain. In previous work, replacement of Mn$^{2+}$ in the water processing PSII by U was shown to influence the entire photosynthetic chain^52^, and this may be the reason for difficulty in identifying a specific PS site that U is attacking. The speciation of U in solution can affect uptake, and precipitation out of solution can occur under certain conditions^13,15,18,20^. With elevated levels of U in solution, there is an increased likelihood of U precipitation, which can negatively impact uptake^21^. Furthermore, as the concentration of uranium approaches and reaches saturation in the system, the uranium and the uranyl ion can form complex polymeric species. Different speciation and complexation of uranium can influence the uptake efficiency and stress response in algae; therefore, the observations in this study may be different than if the algae were exposed to lower concentrations of uranium. Further work is needed to identify how the fluorescence parameters measured via F-IF change when *C. reinhardtii* is exposed to different uranium concentrations.

Although comparing the two photosystems does not provide information about U stress, we observe an increase in the lifetime of the 675-nm and 720-nm peaks upon exposure to U. This implies a decrease in NPQ. Previous work by Vanhoudt et al. observed a similar response upon U exposure to *Arabidopsis thaliana*, along with an increase in photosynthetic efficiency^18^. Despite this being the case, the authors report that U is still toxic to plants, especially with prolonged exposure. The decrease of NPQ is a sign that the carotenoids produced with U-exposure are likely acting as antioxidants against the ROS produced, which is confirmed by the increase in absorption in the UV (250–400 nm), as shown in Fig. 4a,b. The majority of photosynthetic pigments absorb in the range of 400–700 nm; however, ROS such as H$_2$O$_2$ and antioxidant proteins like glutathione and ascorbate absorb in the range of 250–400 nm. Several previous studies that investigated plants’ response to U confirmed that there are increased levels of H$_2$O$_2$, and that glutathione and ascorbate play a significant role in remediating the response produced from U stress^17,23^. Furthermore, Vanhoudt et al. also observed a decrease in the ratio of [Chl]:[Car] in conjunction with the decreased NPQ^18^. Although these authors studied a different plant species, *C. reinhardtii* as a green algae contains similar photosynthetic pigments as higher plants like *Arabidopsis thaliana*. For this reason, it may be possible to have some similar responses between these two species to U-exposure, like decreased NPQ and [Chl]:[Car]. Further work is needed to investigate F-IF of terrestrial plants as both algae and plants such as *A. thaliana* are of interest to remote sensing and environmental contamination monitoring.

Previous work by Herlory et al. measured a decrease in photosynthetic activity and an increase in NPQ in *C. reinhardtii* upon exposure to U in various concentrations after 5 h of exposure^26^. In their study they used PAM fluorometry, and the maximum U concentration was 10$^4$ $\upmu$ g of U/L (or $\sim$25 $\upmu$ M) with a density of 150,000 cells/mL. In the present work, a concentration of 500 $\upmu$ M UO$_2$(NO$_3$)$_2$ is $\sim$1.9$\times$10$^5$ $\upmu$ g of U/L and a cell density of 300,000 cells/mL are used. If we account for the difference in cell density, the work by Herlory et al. would be the equivalent of approximately 50 $\upmu$ M uranium in exposure to the cell density used in this study, *i.e.*, 300,000, making their maximum concentration of U investigated an order of magnitude lower than in this study. The differences in cell concentration, photon flux density, U concentration, and the amount of U taken up by the algae cells can all play a role in the observed differences in measurements. Furthermore, Herlory et al. did not report pigment concentrations, and the concentrations and interactions of the pigments have an impact on the measured fluorescence parameters. Another important distinction between our study and that by Herlory et al. is the analysis method employed. PAM fluorometry measures ChlF kinetics over a period of minutes, whereas fs-LIF or F-IF used in this work interrogates rapid molecular dynamics and energy transfer that occurs on a sub-ns timescale. NPQ is the dissipation of energy from Chl by Car upon energy transfer between the two molecules, which is measurable via ultrafast spectroscopy methods through instantaneous excitation of Chl^43^. Both PAM fluorometry and fs-LIF provide information about NPQ; however, the excitation dynamics are fundamentally different. Further work is needed to determine the limit of detection for U-induced changes in *C. reinhardtii* under similar conditions, as well as comparing the measurements made by F-IF with more established methods such as PAM fluorometry.

This work shows promise for F-IF to be used in rapid and remote environmental monitoring of metal pollution and plant health by allowing for high spatial sensitivity and specificity. Currently, SIF and hyperspectral imaging are the most common techniques for remote plant health monitoring. This technology is more developed than in-field use of filamentation for remote sensing, where both ground-based telescope systems and satellite systems are regularly used for SIF and hyperspectral imaging. Two drawbacks with these latter methods are the large solar-background, which is the result of no temporal gating, and relatively poor spatial resolution. One current technological challenge for filament-supported remote sensing is the limited availability of compact, portable, and vibration-tolerant high-power ultrafast lasers. Recent advances in ultrashort Yb-doped fiber lasers may soon lead to realization of rugged and compact lasers with gigawatt peak powers necessary for in-field filamentation studies^53^. Fiber-based technologies with integrated photonics promise robustness, power scalability and turn-key operation unavailable to the more common Ti:sapphire based systems. Continued progress in source technology will improve the prospects for ultrafast laser filamentation to be employed for environmental monitoring.

## Conclusion

In conclusion, we demonstrate filament excitation of live *C. reinhardtii* algae to be a promising method for remote, in-field monitoring of stress and plant health. We identify optical signatures that can be used to identify U-exposure and investigate the relationship between the pigment content determined from the absorption spectrum and the F-IF measurements to better understand how U influences the plant processes. We find that there is a notable increase in absorbance in the 250–400 nm region for the U-exposed algae. We attribute this to an increase in the production of ROS and other antioxidant-related proteins, such as glutathione and ascorbate, which have been reported to be essential in responding to U stress. This response occurs quickly, as early as one hour after exposure, demonstrating the sensitivity of *C. reinhardtii* to facilitate U detection shortly after exposure. The increased absorbance persists to 24 hours after exposure, when the fluorescence measurements were made in this study. Interestingly, we do not observe signs of cell death, deduced from the observed increase in Chl a concentration with U exposure; however, there is a decrease in [Chl]:[Car], signifying an increase in [Car] which may also be a sign of stress in response production of ROS. The control samples follow the trend of increasing [Car] correlated to a decrease in fluorescence intensity and lifetime due to the NPQ contribution of Car. On the other hand, while the time-integrated intensity decreases with increasing [Car] for U exposure, there is also an increase in fluorescence lifetime, or a decrease in NPQ. The combination of increased [Car] and increased fluorescence lifetime may be a unique response of U stress, allowing it to be distinguished from other stresses; however, further work is needed for confirmation. Lastly, we demonstrate the potential for single-shot remote discrimination of U-exposed algae from the control at a distance of $\sim$35 m, and 125 m with 100 laser shots averaged per time step, or a measurement time of 1.6 s assuming a 1-kHz laser system and comparable data acquisition system. The results of this work are promising for rapid, remote, in-field assessment of plant health and have broader implications for pollution monitoring and nuclear safeguards.

## Methods

### Test species and stress exposure

*C. reinhardtii* algae is obtained from the University of Texas Culture Collection of Algae (UTEX #90). An axenic culture was prepared in Hoagland solution^54^. A small portion of *C. reinhardtii* was transferred from the agar to two 150 mL glass flasks containing 50 mL of Hoagland nutrient growth media using a sterilized metal spatula and are agitated for 1 min twice daily. This results in two primary stock solutions. The flask apertures were sealed with Parafilm and placed under grow lights (390–730 nm) with a photon flux density of 120 $\upmu$ mol/s m$^2$ and a light cycle of 12-h on:12-h off. The algae were propagated at room temperature for 3 weeks before beginning experimental exposure, with a cell density of 300,000 cells/mL.

Previous work demonstrated that U uptake increases when U is in the form of the uranyl ion (UO$_2^{2+}$)^21^ in solutions with lower pH (in the range 4.5–6.5)^15^ and with minimal concentrations of aqueous phosphorous or calcium^13,16^. In order to optimize conditions for U uptake, we prepared a simplified Hoagland nutrient solution following the procedure outlined by Hayek *et al.*^13^. The solution consisted of 0.5 mM MgSO$_4$, 1 mM KCl, 2 mM NH$_4$NO$_3$, 1 mM NaHCO$_3$, and UO$_2$(NO$_3$)$_2\cdot$6 H$_2$O (provided by the Inorganic Materials and Nanomaterials Lab at the University of Nevada Las Vegas) for the U exposure. The concentration of UO$_2$(NO$_3$)$_2$ in solution is 500 $\upmu$ M, and the pH of the solutions was monitored to be at $\sim$5. In order to transfer the algae from the nutrient growth media to the experimental media, we extracted four 5 mL samples from each stock algal media and centrifuge them for 5 min at 3000 rpm to form a pellet. The remaining growth media were disposed, the pellets were washed once in DI water, centrifuged a second time, and resuspended in the experimental media. Two samples from each stock media were resuspended in the control solution (Simplified Hoagland without U) and the remaining two samples from each stock media were exposed to the 500 $\upmu$ M-U in simplified Hoagland solution. There are four total samples per exposure condition: two from stock 1 and two from stock 2 for both the control and uranium-exposed samples. The samples were stored in glass flasks, sealed with parafilm, and agitated twice for 1 minute each over a 24-hour period.

### Steady-state absorption and filament-induced fluorescence

**Figure 8:**
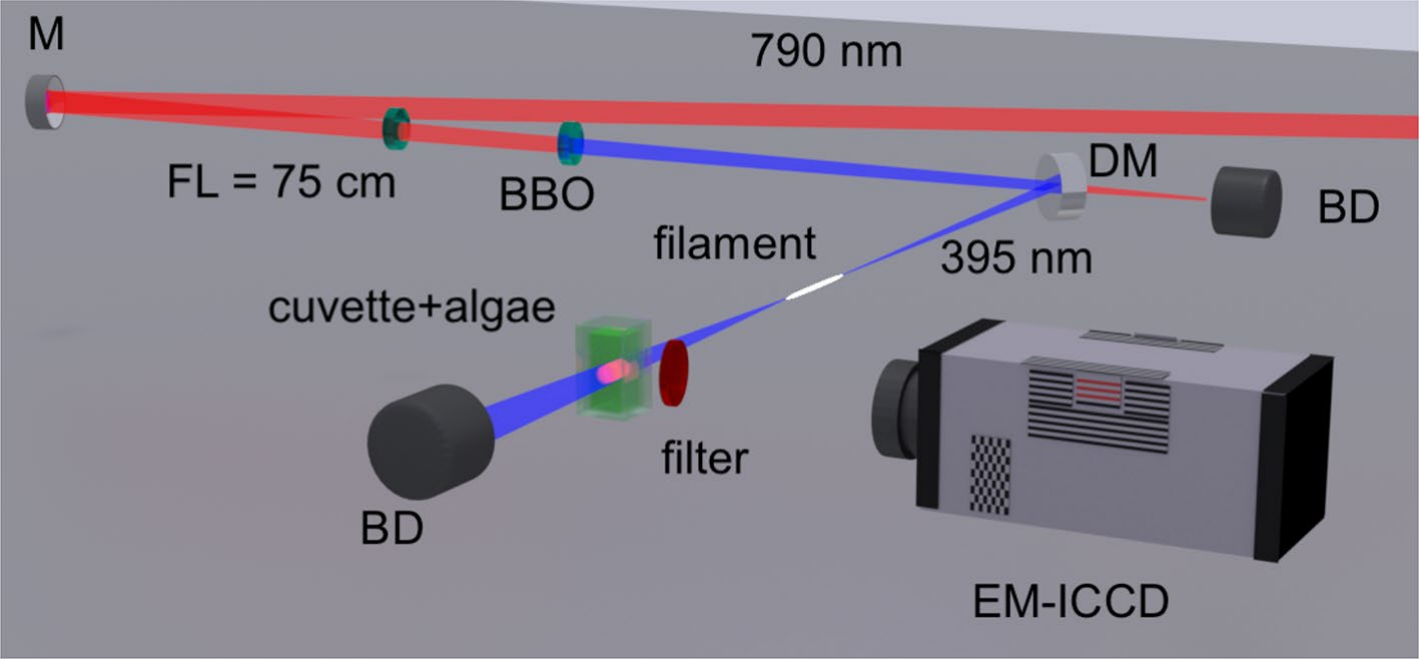
Experimental schematic for the filament-induced fluorescence imaging of algae samples. *BD* beam dump, *DM* dichroic mirror, *FL* focal length, *M* mirror.

Steady-state absorption spectra were recorded with a Varian Cary 50 UV-VIS-NIR spectrophotometer, with a spectral range of 190–1100 nm. Measurements were made 1 hour and 24 hours after stress exposure. The experimental schematic for F-IF is shown in Fig. 8. A Ti:sapphire chirped-pulse amplification system was used, operating at a central wavelength of 790 nm, 480 Hz, 50-fs pulse duration, and an output energy of up to 18 mJ. A half-wave plate was used to control the power of 395-nm light generated after passing through a 200-$\upmu$ m-thick Type I $\upbeta$-Ba(BO$_2$)$_2$ crystal for SHG. A dichroic mirror was used to reject any residual 790-nm light, and the final energy after SHG was $\sim$80 $\upmu$ J. This resulted in a peak power of 1.6 GW, and the critical power for self-focusing at $\sim$395 nm of $\sim$0.3 GW; therefore we have not entered the multiple filament regime which generally requires $\gtrsim$10$\times P_{cr}$^55^. The Gaussian beam diameter was 22 mm, and we seeded the filamentation process with a 75-cm-focal length lens (*f*/30). We placed a 1-cm path length quartz cuvette (Thorlabs, CV10Q35FA) containing the algae samples approximately 50 cm after the termination of filament, resulting in a total excitation distance from the focusing lens of 1.25 m. The filament formed approximately 1 cm prior to geometric focus, and the plasma channel length was $\sim$3 cm as estimated from its optical emission. The F-IF was collected transverse to to the laser propagation direction with an electron multiplying-ICCD (EM-ICCD, PI-MAX4 Princeton Instruments) coupled to a variable telescopic lens. Two filters were used to isolate the main chlorophyll fluorescence bands, one with a central wavelength of 675 nm and a 20-nm-FWHM and another with a central wavelength of 720 nm and a 10-nm-FWHM (Andover). Time-integrated and time-resolved fluorescence measurements were recorded after 24 hours of sample preparation.

## Data Availability

The datasets generated and/or analysed during the current study are available from the corresponding author on reasonable request.
